# Improved referral and survival of newborns after scaling up of intensive care in Suriname

**DOI:** 10.1186/s12887-017-0941-6

**Published:** 2017-11-14

**Authors:** Rens Zonneveld, Natanael Holband, Anna Bertolini, Francesca Bardi, Neirude P. A. Lissone, Peter H. Dijk, Frans B. Plötz, Amadu Juliana

**Affiliations:** 1 Academic Pediatric Center Suriname, Academic Hospital Paramaribo, Flustraat 1, Paramaribo, Suriname; 2 Department of Pathology and Medical Biology, University Medical Center Groningen, University of Groningen, Hanzeplein 1, 9713 GZ Groningen, The Netherlands; 3 Department of Pediatrics, Tergooi Hospitals, Rijksstraatweg 1, 1261 AN, Blaricum, The Netherlands; 4 Department of Pediatrics, University Medical Center Groningen, University of Groningen, Hanzeplein 1, 9713 GZ Groningen, The Netherlands

**Keywords:** NICU, Low-resource setting, Developing country, Neonatal mortality, Suriname

## Abstract

**Background:**

Scaling up neonatal care facilities in developing countries can improve survival of newborns. Recently, the only tertiary neonatal care facility in Suriname transitioned to a modern environment in which interventions to improve intensive care were performed. This study evaluates impact of this transition on referral pattern and outcomes of newborns.

**Methods:**

A retrospective chart study amongst newborns admitted to the facility was performed and outcomes of newborns between two 9-month periods before and after the transition in March 2015 were compared.

**Results:**

After the transition more intensive care was delivered (RR 1.23; 95% CI 1.07–1.42) and more outborn newborns were treated (RR 2.02; 95% CI 1.39–2.95) with similar birth weight in both periods (P=0.16). Mortality of inborn and outborn newborns was reduced (RR 0.62; 95% CI 0.41–0.94), along with mortality of sepsis (RR 0.37; 95% CI 0.17–0.81) and asphyxia (RR 0.21; 95% CI 0.51–0.87). Mortality of newborns with a birth weight <1000 grams (34.8%; RR 0.90; 95% CI 0.43–1.90) and incidence of sepsis (38.8%, 95% CI 33.3–44.6) and necrotizing enterocolitis (NEC) (12.5%, 95% CI 6.2–23.6) remained high after the transition.

**Conclusions:**

After scaling up intensive care at our neonatal care facility more outborn newborns were admitted and survival improved for both in- and outborn newborns. Challenges ahead are sustainability, further improvement of tertiary function, and prevention of NEC and sepsis.

**Electronic supplementary material:**

The online version of this article (10.1186/s12887-017-0941-6) contains supplementary material, which is available to authorized users.

## Background

Neonatal mortality in developing countries continues to be a chief global health challenge [1, 2]. A recent global report indicates that over 40% reduction of neonatal mortality can be achieved by implementation of institutional care in lower resource countries [3]. In particular, local or regional neonatal care facilities with integrated availability of perinatal and neonatal intensive care can reduce mortality [4]. For example, newborns born in a rural hospital featuring a neonatal intensive care unit (NICU) in Uganda were almost twice as likely to survive than those born outside [5]. Moreover, introduction of a neonatal care facility in a low-income district in India reduced neonatal mortality rate (NMR) by 21% after the first two years [6]. Improving interventions within existing neonatal care facilities (e.g., training of personnel, refurbishment, infection prevention) can improve mortality and enhance tertiary function for newborns in need of intensive care [6–9].

In Suriname NMR in 2009 was 16.0 per 1000 live births. However, detailed data on demographics and outcomes of newborns are lacking. In 2008 the neonatal care facility at the Academic Hospital Paramaribo (AHP), which also incorporated the first and only NICU in Suriname, opened its doors. The ability to treat premature and critically ill newborns was an important step towards reducing mortality. At the end of March 2015 the facility moved to a new and modern environment. This transition solidified availability of neonatal intensive care in Suriname with reinforcement and training of personnel, new equipment, continuous availability of supplies, and protocol-based care. Since this facility is the only referral center for newborns requiring intensive care in Suriname, morbidity and mortality of newborns treated here reflect their outcomes at the national level.

Therefore, as a benchmark for future investigations, we developed a registry to describe demographics and outcomes of newborns admitted to the neonatal care facility. Additionally, to evaluate the impact of improvements we compare referral pattern, mortality and morbidity of newborns treated in periods before and after the transition. Ultimately, this could lead to better prospective registry and care for critically ill newborns in Suriname.

## Methods

### Study design

We performed a retrospective (pre-and post transition) study in the neonatal care facility of the AHP during the periods July 1st 2014 to March 29th 2015 (Period 1) and March 31st to December 31st 2015 (Period 2). The impact of the transition was described by analyzing demographics and outcomes of all inborn and outborn newborns admitted within these two periods. Excluded were newborns whom were treated in both periods and of whom insufficient information (i.e., no or incomplete paper charts) was available to confirm outcomes. We received a waiver from our institutional ethical board.

### Setting and interventions

Suriname is a small middle-income country with a multiethnic society and has an annual birth rate of about 10,000 births. Over 90% of births take place at delivery rooms of one of four hospitals situated in Suriname’s capital Paramaribo (inhabited by more than half of Suriname’s population). About 30% of births take place at the delivery room of the AHP. The neonatal care facility at the AHP serves as the only referral hospital for critically ill newborns. Since the opening in 2008, between 350 and 400 newborns are treated each year in one room with 12 beds, with NICU capacity operating at Level III [9]. Newborns are generally only actively treated with a birth weight (BW) ≥ 750 g and/or gestational age (GA) ≥ 27 weeks.

On March 30th 2015 the facility moved to a completely new, modern and spacious environment with central climate control and new equipment (i.e., ventilators, incubators, air-humidifiers, ultrasound machines and multi-parameter monitors). Capacity for mechanical ventilation and continuous positive airway pressure (CPAP) was doubled. The NICU (6 beds), high care (HC) (6 beds), and medium care (MC) (4 beds) capacity in the new facility remained the same until February 2016 (when a separate space for the MC was opened and the NICU capacity increased to 10 beds).

Total expense for the new building and equipment was 2.6 million US dollars. Funds were collected from kind donations from governmental and private organizations and from Surinamese companies. Since there were no architects or contractors available within Suriname with experience in designing a NICU level neonatal care facility, we relied on guidelines from developed countries and local creativity and practical experience to realize the project within budget, without the need for expensive consultants. For example, one of the savings came from using venturi mechanism based suction devices powered by compressed air, avoiding the need for a separate central vacuum system.

Admission criteria remained the same. Obstetric nurses were trained in neonatal life support and the number of residents in the obstetric and pediatric department was increased. For both day and evening shifts a separate resident was assigned to the NICU exclusively. Shortly before the transition, nurses were trained in intensive neonatal care and their number was expanded to 1 per 3 or 4 beds. New charts for vital signs, ventilation settings, and fluid management were implemented. A breast-feeding and nutrition program was started to help reduce cases of necrotizing enterocolitis (NEC) and mothers were allowed at the bedside twice as long as before. Systematic infection prevention (i.e., stringent guidelines and more facilities for hand washing, providing of patient specific (disposable) materials, Extended Spectrum Beta-Lactamase (ESBL) outbreak control) was enforced.

### Data collection and analysis

Data were collected from paper medical records on maternal, obstetric and perinatal history, birth location, reason for admission, hospital course, and outcomes. A single major cause of death was determined. For each included newborn we determined the highest level of care during their stay by assigning criteria for NICU, HC or MC retrospectively according to local protocol (Additional file 1: Table S1). Primary outcome was mortality: NMR at the AHP and at the neonatal care facility divided in early (i.e., in-hospital death before 7 days of life) and late (i.e., in-hospital death of at term newborns after 7 days of life), GA-specific mortality, BW-specific mortality, and cause-specific mortality. Secondary outcomes were highest level of care, respiratory treatments (CPAP, mechanical ventilation, surfactant), use of antibiotics, development of respiratory complications, i.e., pneumothorax, bronchopulmonary dysplasia (BPD; i.e., oxygen dependence >28 days of age), ventilator-associated pneumonia (VAP; i.e., positive tracheal aspirate culture after ventilation), development of NEC and sepsis (i.e., early (<72 h after birth) and late (>72 h after birth) onset clinical (i.e., clinical suspicion, treated with antibiotics for 7 days, raised c-reactive protein levels)) and blood culture positive sepsis, blood and ESBL culture results, and duration of stay.

### Statistical analysis

Incidence rates and epidemiological determinants were calculated for the inclusion period. Categorical variables are presented as numbers and percentages with 95% confidence intervals (CI) and continuous variables as means with standard deviations (SD) or, if not normally distributed, as medians with ranges. Continuous variables were compared with a student t-test and categorical variables were compared with Chi-Square. Relative risk (RR) and 95% CI were calculated. *P*-values <0.05 were considered statistically significant.

## Results

### Demographics and referral

A total of 626 newborns were treated at the neonatal care facility of whom 601 (320 before and 281 after the transition) were included (Table 1). Overall demographics were comparable between both periods, with similar percentages of missing data, showing high prevalence of (antenatal) risk factors for mortality and morbidity (Table 1). In period 2 significantly more outborn newborns (RR 2.02; 95% CI 1.39–2.95; *P* < 0.001) were treated with similar mean birthweight (2183 ± 845 g vs. 1915 ± 990 g; *P* = 0.16). Prematurity was the main reason for admission for all inborn (48.3%; 95% CI 44.0–52.7) and outborn (66.0%; 95% CI 56.3–74.5) newborns, followed by respiratory distress and suspected infection (Table 1).

**Table 1:** Demographics of newborns admitted to the neonatal care facility before and after the transition

		Period 1 (July 2014–March 2015)		Period 2 (April 2015–December 2015)	
		N	% (95% CI)	N	% (95% CI)
Live births	Total at AHP	2353		1972	
Admissions to facility	Total	331		295	
	Included	320	96.7	281	95.3
	Inborn	284	88.7 (84.8–91.8)	217	77.2 (72.0–81.7)
	Outborn^b^	36	11.3 (8.2–15.2)	64	22.8 (18.3–28.0)
Maternal age (Years)	<20	54	16.9 (13.2–21.4)	36	12.8 (9.4–17.2)
	20–34	168	52.5 (47.0–57.9)	140	49.8 (44.0–55.6)
	≥35	46	14.4 (11.0–18.6)	24	8.5 (5.8–12.4)
	Missing	52	16.3	81	28.8
Pregnancy	HIV	6	1.9 (0.9–4.0)	2	0.7 (0.2–2.6)
	Diabetes	18	5.6 (3.6–8.7)	20	7.1 (4.7–10.7)
	PIH / Preeclampsia	60	18.8 (14.9–23.4)	62	22.1 (17.6–27.3)
	Antenatal steroids^c^	47	46.1 (36.7–55.7)	55	53.9 (44.3–63.3)
	Infection risk^d^	47	14.7 (11.2–19.0)	38	13.5 (10.0–18.0)
Mode of delivery	Vaginal	187	58.4 (53.0–63.7)	167	59.4 (53.6–65.0)
	Caesarean section	105	32.8 (27.9–38.1)	94	33.5 (28.2–39.2)
	Missing	28	8.8	20	7.1
Sex	Male	162	50.6 (45.2–56.1)	155	55.2 (49.3–60.9)
	Female	158	49.4 (43.9–54.8)	126	44.8 (39.1–50.7)
Gestational age (Weeks)	<28	16	5.0 (3.1–8.0)	13	4.6 (2.7–7.8)
	28–32	48	15.0 (11.5–19.3)	47	16.7 (12.8–21.5)
	33–36	114	35.6 (30.6–41.0)	100	35.6 (30.2–41.3)
	≥37	132	41.3 (36.0–46.7)	110	39.1 (33.6–45.0)
	Missing	10	3.1	11	3.9
Birth weight (Grams)	<1000	26	8.1 (5.6–11.6)	23	8.2 (5.5–12.0)
	≥1000–1499	48	15.0 (11.5–19.3)	33	11.7 (8.5–16.0)
	≥1500	242	75.6 (70.6–80.0)	221	78.6 (73.5–83.0)
	Missing	4	1.3	4	1.4
Apgar Score at 5’	<5	24	7.5 (5.1–10.9)	7	2.5 (1.2–5.1)
	Missing	45	14.1	47	16.7 (12.8–21.5)
Ethnicity	Maroon	87	27.2 (22.6–32.3)	72	25.6 (20.9–31.0)
	Creole	85	26.2 (22.0–31.7)	72	25.6 (20.9–31.0)
	Hindo-Surinamese	59	18.4 (14.6–23.1)	55	19.6 (15.4–24.6)
	Javanese	15	4.7 (2.9–7.6)	21	7.5 (4.9–11.2)
	Amerindian	10	3.1 (1.7–5.7)	7	2.5 (1.2–5.1)
	Chinese	2	0.6 (0.2–2.2)	2	0.7 (0.2–2.6)
	Other^e^	31	9.7 (6.9–13.4)	32	11.4 (8.2–15.6)
	Missing	31	9.7	20	7.1
Initial reason for admission^a^	Prematurity	152	47.5 (42.1–53.0)	148	52.7 (46.8–58.4)
	Respiratory distress^f^	119	37.2 (32.1–42.6)	122	43.4 (37.7–49.3)
	Suspected infection^g^	91	28.4 (23.8–33.6)	97	34.5 (29.2–40.3)
	Perinatal asphyxia^h^	39	12.2 (9.0–16.2)	30	10.7 (7.6–14.8)
	Congenital malformations^i^	42	13.1 (9.9–17.3)	35	12.5 (9.1–16.8)
	Other^j^	71	22.2 (18.0–27.1)	49	17.4 (13.4–22.3)

*AHP* Academic Hospital Paramaribo, *NICU* neonatal intensive care unit, *HC* high care, *MC* medium care, *PIH* pregnancy-induced hypertension, *RDS* respiratory distress syndrome

^a^ Newborns could have more than one reason for admission

^b^ Includes: delivery rooms of four other hospitals in Paramaribo and one other hospital in Nickerie, birth clinics in rural and interior parts of Suriname, and home births

^c^Administered in two doses of dexamethasone in the case of suspected premature birth before GA of 34 weeks (calculated for a total of *N* = 102 newborns in period 1 and N = 102 in period 2)

^d^Includes: premature rupture of membranes (PROM), intrapartum fever and/or antibiotics, positive maternal Group-B streptococcus culture

^e^Includes: Caucasian, Brazilian, or mixed

^f^Includes: neonatal respiratory distress syndrome, congenital pneumonia, pulmonary hemorrhage, pneumothorax, meconium aspiration syndrome, and transient neonatal tachypnea

^g^Includes: newborns defined with clinical symptoms of infection by admitting physician

^h^Includes: asphyxia defined by admitting physician (e.g., in the case of either need for resuscitation or Apgar <5 beyond 5 min; lactate acidosis with base excess <16; coma or seizures after birth; findings with cerebral ultrasound such as edema)

^i^Includes: diaphragmatic hernia, congenital heart defects, gastro-intestinal anomalies and neurological malformations

^j^Includes: hypoglycemia, dysmaturity, jaundice, and social indications

### Mortality

NMR of inborn newborns born at the AHP was lower in period 2 (*P* = 0.02) (Table 2). After the transition, reduction in mortality was greatest in newborns treated at NICU level care (*P* < 0.01), with a GA above 28 weeks (RR 0.42; 95% CI 0.25–0.72; *P* = 0.002), and outborn newborns (P = 0.02). A trend in decrease in mortality was observed in late mortality (*P* = 0.06), inborn newborns (*P* = 0.07), and in newborns with a birth weight (BW) above 1500 g (P = 0.07). A significant reduction in mortality was observed in cases of sepsis (*P* = 0.01) and perinatal asphyxia (*P* = 0.03). Sepsis was the main cause of death in period 1 (34.5%; 95% CI 23.4–47.7), and second in period 2 (26.7%; 95% CI 14.2–44.4). For newborns with a BW < 1000 g late-onset sepsis was the main cause of death in both periods (44.8%; 95% CI 28.4–62.5).

**Table 2:** Mortality of newborns treated at the facility before and after the transition

		Period 1 (*N* = 320) (July 2014–March 2015)		Period 2 (*N* = 281) (April 2015–December 2015)		Relative Risk (95% CI)	*P*-value
		N	%	N	%		
Overall mortality	Total at AHP (per 1000 live births)^a^	23.4		13.2		0.56 (0.36–0.90)	0.02
	Total at facility	55/320	17.2	30/281	10.7	0.62 (0.41–0.94)	0.02
	Total early neonatal mortality	29/320	9.1	18/281	6.4	0.70 (0.40–1.24)	0.23
	Total late neonatal mortality	26/320	8.1	12/281	4.3	0.53 (0.27–1.02)	0.06
	Inborn	42/284	14.8	20/217	9.2	0.62 (0.38–1.03)	0.07
	Outborn	13/36	36.1	10/64	15.6	0.43 (0.21–0.89)	0.02
	Newborns with NICU level care	52/159	32.7	29/172	16.9	0.52 (0.35–0.77)	<0.01
Gestational age-specific mortality	<28 weeks	6/16	37.5	8/13	61.5	1.64 (0.76–3.53)	0.20
	28–32 weeks	12/48	25.0	5/47	10.6	0.43 (0.16–1.11)	0.08
	33–36 weeks	14/114	12.3	4/100	4.0	0.33 (0.11–0.96)	0.04
	≥37 weeks	20/132	15.2	8/110	7.3	0.48 (0.22–1.05)	0.07
	Missing	3		5			
Birth weight-specific mortality	<1000 g	10/26	38.5	8/23	34.8	0.90 (0.43–1.90)	0.79
	≥1000–1499 g	13/48	27.1	6/33	18.2	0.67 (0.28–1.59)	0.36
	≥1500 g	30/242	12.4	16/221	7.2	0.58 (0.33–1.04)	0.07
	Missing	2		0			
Cause-specific mortality	Sepsis^b^	19/96	19.8	8/109	7.3	0.37 (0.17–0.81)	0.01
	Early-onset sepsis	10/44	22.7	3/59	5.1	0.22 (0.07–0.77)	0.02
	Late-onset sepsis	9/52	17.3	5/50	10.0	0.58 (0.21–1.61)	0.29
	Perinatal asphyxia	12/38	31.6	2/30	6.7	0.21 (0.51–0.87)	0.03
	Prematurity complications^c^	*7/*157	4.5	5/148	3.4	0.76 (0.25–2.34)	0.63
	Congenital malformations^d^	12/42	28.6	9/35	25.7	0.90 (0.43–1.88)	0.78
	Other^e^	5		6			

*AHP* Academic Hospital Paramaribo, *NICU* neonatal intensive care unit

^a^Including deaths at the delivery room (13 before and 6 after the transition)

^b^Includes: newborns with clinical suspicion, treated with antibiotics for 7 days, raised c-reactive protein levels, and positive blood culture

^c^Includes: respiratory insufficiency or pneumothorax with RDS and extreme prematurity, necrotizing enterocolitis; intraventricular hemorrhage

^d^Includes: diaphragmatic hernia, congenital heart defects, gastro-intestinal anomalies and neurological malformations

^e^Includes: persistent pulmonary hypertension of the neonate (PPHN), pneumothorax, cardiac tamponade, and kernicterus

### Treatments and morbidity

Based on our criteria (Additional file 1: Table S1) significantly more NICU level care was given in period 2 (*P* < 0.01) (Table 3). More mechanical ventilation and surfactant were applied after the transition. No difference in prevalence of VAP or pneumothorax was observed and there was a trend in increases incidence of BPD (*P* = 0.07) (Table 4). Grade 2 or higher NEC was present at high incidence in newborns with a BW < 1500 g in both periods (5.4% and 12.5%, respectively). Sepsis (either early or late-onset) was prevalent in over 30% of patients in both periods, of which half was LOS. During both periods, outbreaks with ESBL bacteria led to a significant prevalence of ESBL positive cultures.

**Table 3:** Trends in treatments at the facility in two time periods

		Period 1 (N = 320) (July 2014–March 2015)		Period 2 (N = 281) (April 2015–December 2015)		Relative Risk (95% CI)	*P*-value
		N	%	N	%		
Highest level of care^a^	NICU	159	49.7	172	61.2	1.23 (1.07–1.42)	<0.01
	HC	75	23.4	60	21.4	0.91 (0.68–1.23)	0.54
	MC	86	26.9	49	17.4	0.65 (0.47–0.87)	<0.01
Respiratory treatment	CPAP	100	31.3	106	37.7	1.21 (0.97–1.51)	0.10
	Mechanical ventilation	38	11.9	55	19.6	1.65 (1.13–2.41)	0.01
	Surfactant	15	4.7	21	7.5	1.59 (0.84–3.03)	0.16
Antibiotics received	Total	173	54.1	170	60.5	1.12 (0.97–1.29)	0.11

*NICU* neonatal intensive care unit, *HC* high Care, *MC* medium Care, *CPAP* continuous positive airway pressure

^a^Determined with local criteria given in Additional file 1: Table S1

**Table 4:** Morbidity of newborns treated at the facility in two time periods

		Period 1 (N = 320) (July 2014–March 2015)		Period 2 (N = 281) (April 2015–December 2015)		Relative Risk (95% CI)	*P*-value
		N	%	N	%		
Respiratory morbidity	BPD	4	1.3	10	3.6	2.85 (0.90–8.98)	0.07
	VAP	9	2.8	5	1.8	0.63 (0.21–1.87)	0.41
	Pneumothorax	4	1.3	7	2.5	1.99 (0.59–6.74)	0.27
NEC^a^	Total	10	13.5	12	21.4	1.59 (0.74–3.40)	0.24
	≥ Stage 2	4	5.4	7	12.5	2.31 (0.71–7.51)	0.16
Sepsis^b^	Total	96	30.0	109	38.8	1.29 (1.03–1.62)	0.02
	Positive blood culture	38	11.9	25	8.9	0.75 (0.46–1.20)	0.24
Positive ESBL culture^c^	Total	34	10.6	39	13.9	1.31 (0.85–2.01)	0.22
Duration of stay (days)		Mean	SD	Mean	SD		
		13	16	14	18		0.44

*BPD* bronchopulmonary dysplasia, *VAP* ventilator-associated pneumonia, *NEC* necrotizing enterocolitis, *ESBL* extended spectrum beta-lactamase

^a^Calculated for newborns with a birthweight below 1500 g (*N* = 74 and *N* = 56 in period 1 and period 2, respectively)

^b^Includes: early and late-onset clinical (i.e., high clinical suspicion, treated with antibiotics for 7 days; raised C-reactive protein levels) and blood culture positive sepsis

^c^Includes: blood and urine cultures and cultures on (tracheal aspirate, skin and anal) swabs, central lines or ventilation tubes

## Discussion

Improvements at the neonatal care facility led to an increase of newborns that received intensive care with a significant reduction in their mortality. Furthermore, newborns with a GA above 28 weeks and/or BW ≥ 1500 g showed a significantly reduced mortality rate. A striking reduction in mortality was seen in cases of perinatal asphyxia and sepsis. In addition, after the transition a two-fold increase in admission of outborn newborns, with similar demographics and increased survival rates, was observed. These findings indicate enhanced tertiary function and centralization of neonatal intensive care in Suriname, which may play a significant role in reducing neonatal mortality in Suriname.

Other studies performed in developing countries have shown similar patterns in improvement of mortality after scaling up of neonatal care facilities. Creation of a level II sick newborn care unit (SNCU) (i.e., with introduction of bed warmers and central oxygen) in a district hospital in India led to a significant reduction of regional NMR of mostly newborns with a BW < 1500 g [6]. Another pre-and-post intervention study in India showed that basic interventions (i.e., promotion of enteral nutrition, asepsis regulations and training of nurses) led to an immediate and stable reduction of NMR and birth-weight specific survival of newborns with a BW < 1500 g, but not with a BW < 1000 g, primarily after reduced incidence and mortality of sepsis [7]. Introduction of nasal CPAP at a NICU in Nicaragua reduced mortality amongst total newborns receiving ventilation assistance (i.e. either mechanical ventilation or CPAP) [8]. Improvement (i.e., new equipment, refurbishment and training of personnel) of a newborn unit to a Level III NICU at a teaching hospital in Ghana led to significant reduction of mortality amongst newborns with a BW < 2500 g, mostly secondary to significantly reduced incidence of perinatal asphyxia [9].

In these studies, training and expansion of personnel was a universal denominator for improvement of care, which was also part of our intervention. Systematic training of midwives in neonatal resuscitation has been a challenge in low resource countries and so far has yielded positive results only in low risk settings, and takes time with need for strong re-enforcement and repetition before an effect on neonatal mortality is observed [10–12]. However, increasing the number of nurses per infant at the NICU may have a beneficial effect on neonatal outcome [13, 14]. Further improvement of survival may then be accomplished with increased capacity for neonatal intensive care (e.g., increased capacity for (modernized) ventilation). We observed a significant increase of use of neonatal intensive care commodities in the post-transition period. Indeed, both higher level and volume of neonatal intensive care have been associated with better survival of newborns with a BW < 1500 g [15, 16]. While this seems an intuitive and logical effect, it is important to realize that positive effects of higher capacity can only be sustained with continuous and balanced availability of trained personnel, which can be challenging in the lower resource setting [17, 18]. Illustratively, in our population the reduction of admission rates in the post transition period coinciding with increased number of nurses per bed may have been beneficial for survival. However, the amount of nurses per infant at our facility is still less than recommended for the intended level of care (i.e., one nurse per one or two beds), which may partially explain our finding that the mortality rate in the most vulnerable small preterm infants (i.e., with a BW < 1000 g and <28 weeks of GA) did not decrease [19]. However, restricting the number of beds in case of understaffing is extremely difficult when there are no other NICU level referral options in Suriname.

Admission of more outborn neonates indicates an enhanced regional function of our neonatal care facility, which was shown to be beneficial for their survival depending on the referral system. In Ghana, survival of outborn newborns at the refurbished NICU was only beneficial to those referred from private health facilities [9]. In our population, outborn newborns, mostly referred from birth clinics and private or public Level II SNCUs at other hospitals, died more frequently than inborn ones in both periods. Delays in transfer or higher prevalence of antenatal (e.g., preeclampsia) and neonatal (e.g., prematurity) risk factors could have contributed to this [20–22]. However, the fact that in our study demographics of outborn newborns were similar in both periods indicates that better survival after the transition was mostly due to enhanced neonatal intensive care, independent of presence of antenatal and neonatal risk factors. Screening regimens for antenatal risk factors at surrounding birth clinics and in-utero transfer to our birth clinic, thereby creating proximity to our neonatal care facility, could further enhance tertiary function and improve survival in Suriname [23, 24].

Mortality due to both perinatal asphyxia and sepsis were reduced in the post transition period. For inborn newborns, training of obstetric nurses may have contributed to the reduction in mortality of sepsis and similarly to less cases and better outcome of asphyxia. Additionally, for both inborn and outborn newborns efficient treatment (e.g., modern equipment for mechanical ventilation or circulatory support) at our refurbished NICU could have had beneficial effect on survival of both. In the case of late-onset sepsis, incidence and mortality remained the same after the transition. This indicates that our asepsis interventions, aimed primarily at prevention of transmission of pathogens, failed, which is also reflected in similar amounts of ESBL-positive blood cultures among both study periods. These results stress that in our setting strict enforcement of asepsis protocol remains challenging, but should be prioritized.

Mortality of newborns with a BW < 1000 g remained high after the intervention. High mortality of newborns with a BW < 1000 g was also observed in earlier reports in a Level II SNCU in Jamaica, a Level III neonatal care facility in South Africa and at multiple NICUs in Brazil and around the world [1, 25–27]. In our low-resource setting, the fact that these newborns demand a disproportionate share of scarcely available human and non-human recourses is a significant limitation for improvement. However, almost half of them died of late-onset sepsis, indicating that more effective infection prevention, including antibiotic stewardship, might substantially increase their survival rates. Additionally, a major cause for morbidity amongst newborns with a BW < 1500 g in our study was NEC (Table 4). Prevalence of NEC remained high, despite promotion of feeding with human breast milk. Recent evidence from NICUs in developed countries has shown that simple interventions (i.e., early human milk feedings, rigorous feeding protocol and restricted feeding during indomethacin treatment and blood transfusions, and selective antibiotic usage) can reduce incidence of NEC [28]. These interventions are cost-effective and can also easily be applied in lower resource settings [29]. A major limitation in our setting is the unavailability of total parenteral nutrition, but at the same time the low adherence to breast milk offers a major opportunity for improvement. Lastly, the increased number of cases of NEC, along with the increase in incidence of BPD, may be the unfortunate effect of more intensive care (e.g., more ventilation, more early antibiotics) and better survival.

Limitations to this study were missing data (e.g., scarce data on additional outcomes such as intraventricular hemorrhage, retinopathy of the premature, post-discharge survival), the retrospective nature of this study, and relatively small numbers for complications with a low incidence. Although we collected data to determine the highest level of care, we were not able to apply an index to indicate severity of disease of newborns.

## Conclusions

This study shows that scaling up of neonatal intensive care in Suriname substantially reduced mortality of both in and outborn newborns through its enhanced availability and centralization. Challenges ahead are sustainability, further improvement of tertiary function, and prevention of sepsis and NEC with implementation of cost and resource effective interventions.

## Additional file

Additional file 1: Table S1. Local criteria for medium, high or intensive care (DOCX 14 kb)

## Abbreviations

AHP: Academic Hospital Paramaribo
BPD: Bronchopulmonary dysplasia
BW: Birth weight
EOS: Early onset sepsis
ESBL: Extended spectrum beta-lactamase
GA: Gestational age
LOS: Late onset sepsis
Maroon: Descendant from Africans that escaped slavery and established independent societies (e.g., term predominantly used in South America and on Caribbean Islands)
NEC: Necrotizing enterocolitis
NICU: Neonatal intensive care unit
NMR: Neonatal mortality rate (i.e., number of neonatal deaths per 1000 live births
SNCU: Sick newborn care unit
VAP: Ventilator-associated pneumonia
