# Supplementary material for: Improved referral and survival of newborns after scaling up of intensive care in Suriname
Source: BMC Pediatr. 2017 Nov 14;17:189. doi: 10.1186/s12887-017-0941-6 (PMC5686851; doi:10.1186/s12887-017-0941-6)
Supplement: Additional file 1: Table S1. — Local criteria for medium, high or intensive care (DOCX 14 kb) [file 12887_2017_941_MOESM1_ESM.docx]

**Table S1:** Local criteria for medium, high or intensive care

| **Criteria** | **Medium care** | **High care** | **Intensive care** |
| --- | --- | --- | --- |
| **Gestational age** (weeks) | > 37 | 32-37 | <32 |
| **Birth weight** (grams) | >2000^1^ | 1000-2000 | <1000 |
| **Respiratory** | None | Nasal oxygen cannula | Mechanical ventilation  CPAP |
| **Circulatory** | None | Peripheral vein cannula | Arterial line  Cardiotonics |
| **Gastro-intestinal** | Complete oral feeding | Tube feeding | Parenteral feeding  Gastro-intestinal surgery |
| **Metabolic** | Phototherapy (at term)  Hypoglycemia | Phototherapy |  |
| **Hematologic** |  | Blood transfusion | Thrombocytes, FFP  Exchange transfusion |
| **Neurologic** |  |  | Seizures  Perinatal asphyxia  Cerebral ultrasound |
| **Infection** | Observation after maternal risk factors^2^ |  | Sepsis, meningitis, pneumonia |
| **Other** |  |  | Congenital heart defects |

CPAP = continuous positive airway pressure; FFP = Fresh Frozen Plasma.

^1^ At term dysmaturity was also a criteria for admission to the MC;

^2^ Prolonged rupture of membranes (PROM), intrapartum fever and/or antibiotics, positive maternal Group-B streptococcus culture.
